# Sleep Data, Physical Performance, and Injuries in Preparation for Professional Mixed Martial Arts

**DOI:** 10.3390/sports7010001

**Published:** 2018-12-20

**Authors:** Corey A. Peacock, Mauricio Mena, Gabriel J. Sanders, Tobin A. Silver, Douglas Kalman, Jose Antonio

**Affiliations:** 1 Department of Health and Human Performance, Nova Southeastern University, Fort Lauderdale, FL 33328, USA; mm3932@nova.edu (M.M.); tsilver@nova.edu (T.A.S.); dkalman@nova.edu (D.K.); ja839@nova.edu (J.A.); 2 Department of Kinesiology and Health, Northern Kentucky University, Highland Heights, KY 41099, USA; sandersg1@nku.edu

**Keywords:** health, professional athlete, mma, injury

## Abstract

The purpose of this investigation is to present observational data regarding sleep variables in professional Mixed Martial Arts (MMA) athletes. These sleep performance measures were related to physical performance and injury in MMA athletes. Eight professional athletes were placed into a quasi-controlled, multivariable fight-camp environment for a six-week period in preparation for fight competition. Throughout a six-week fight camp environment, athletes were continuously monitored for sleep performance measures (sleep latency, sleep efficiency, onset, and wake variances) via validated wearable sleep monitoring technology. Athletes were tested seven days prior to competition on measures of physical performance (vertical jump, VO_2_max, heart rate recovery, prowler sled push, and pull-ups). Multiple correlational analyses were utilized to assess relationships between all sleep and physical performance measures. There were significant (P < 0.05) correlations between sleep latency and VO_2_max, heart rate recovery, prowler sled push, vertical jump, and missed practice sessions. There were also significant (P < 0.05) correlations between average fall asleep time and heart rate recovery. Lastly, there were significant (P < 0.05) correlations between sleep efficiency, heart rate recovery, and missed practice sessions. MMA athletes who exhibited consistency in sleep demonstrated stronger relationships with performance testing during the fight-camp period.

## 1. Introduction

Research has shown that greater than seven hours of sleep are necessary for proper human function and recovery [1]. Sleep is essential for maintaining normal homeostasis [2]. Sleep-wake cycles are critical in human circadian rhythms. Their disruption may have detrimental consequences regarding behavior and performance [3]. The harmonious co-existence of distinct circadian rhythms cannot be assumed when the normal sleep-wake cycle is disrupted [4]. Disruptions occur due to busy schedules that do not allow for normal sleep patterns, expeditious travel across multiple time-zones, and extreme athletic or recreational pursuits where sleep is restricted or deprived altogether [3]. Continuous research has shown that sleep deprivation has been associated with longer reaction times and reduced force [4,5]. Sleep deprivation strongly correlates with increased lapsing, cognitive slowing, memory impairment, and decreased vigilance [6,7,8,9,10,11,12]. Brief arousal during sleep, timing of sleep, and wakefulness correlate directly with daytime performance and academics [13,14,15].

The effects of sleep deprivation or an inadequate amoujnt of sleep (less than 7 h in the preceding 24 h) are evident in the athletic population [16]. This is apparent when analyzing the susceptibility of travelling sport teams who are losing sleep due to jet lag [16]. Yet, sleep deprivation of up to at least 24 h has not shown influence in physical performance capabilities like muscular strength, cardiovascular response, or respiratory response [17,18]. In spite of being able to overcome the disadvantageous effects of sleep loss in a single-day effort, trying to maintain peak performance in sustained-repeated exercise bouts and on consecutive days might not be feasible. Research indicates that sub maximal lifting tasks are affected more by sleep loss than maximal efforts, especially for the first two nights of sequential sleep restriction [19]. The detrimental effects of accumulated fatigue due to sleep loss become predominant during training sessions [19].

Research has shown that physical activity increases the dissociation between subjective sleepiness and objective performance levels during extended wakefulness in humans [20,21]. The literature supports the notion that exercise during a prolonged period of wakefulness leads to an increased risk in human error. However, the effects of exercise on cognitive and motor performance while exposed to sleep deprivation are unclear [20,21].

There are still unanswered questions with regard to the sleep necessities of professional athletes. As previously mentioned in anaerobic, traveling sports such as the American Football and Powerlifting, they have displayed how sleep can affect athletic pefromance [16,19]. Currently, there is an emergent professional sport known as MMA (Mixed Martial Arts), in which athletes utilize the anaerobic energy systems and travel for competiton. MMA is a combat sport, which incorporates a wide range of grappling, kicking, and punching techniques from several disciplines such as jiu-jitsu, boxing, kickboxing, judo, taekwondo, and karate. MMA athletes utilize training camp formats with the purpose of competition preparation. Because of the great amount of sleep data and its lack in the sport of MMA, the purpose of this investigation is to present observational data with regard to sleep and physical performance in professional MMA athletes. It was hypothesized that consistent sleep metrics will relate to improved performance and health in professional MMA athletes. 

## 2. Materials and Methods

### 2.1. Participants

The multivariable study model included eight professional, male, Mixed Martial Artists (27.7 ± 3.4 years, 5 Brazilian, and 3 African-American) entering fight-camp preparation. This “real-world” observational study was conducted over a six-week period, and consisted of physical performance testing, tactical practices, strength and conditioning sessions, and competition. The inclusion criteria were professional medical clearance (blood-work, physical examination, health history) and MMA fighters signed with and working for the Ultimate Fighting Championship (UFC) professional organization. The exclusion criteria included athletes unable to pass medical examinations and drug testing.

The subjects were placed in a quasi-controlled environment for the six-week competition period in which practice schedule, promotional appearances, and competition was provided by coaching and managerial staff. Fighters practiced 1–2 times per day, tested for physical performance measures, and competed in a Mixed Martial Arts fight competition during this period. Athletes were continuously monitored throughout the duration of the six-week period for sleep performance measures. The performance measures were assessed as a battery of tests seven days prior to the competition. The Nova Southeastern University Institutional Review Board approved this report of data in the profession of mixed martial arts and all athletes provided consent to participate in the six-week competition period.

### 2.2. Protocol Physical Performance Measures

All athletes were tested for the following physical performance measures: maximum muscular power/strength, cardiovascular performance, and endurance. Athletes were also tracked for the total number of injuries and missed practices due to injury.

- Vertical Jump—The athletes performed the vertical jump using a commercial vertec device (Sports Imports, Columbus, OH, USA). After using the stack of adjustable horizontal vanes to determine the flat-footed standing reach, the stack of vanes was raised to an estimated height so that the athletes were capable of reaching the lowest set of vanes but incapable of reaching the highest vane. After the athletes generated power and jumped as high as possible vertically, the difference between standing reach and vertical reach was computed. The highest vertical difference trial was utilized as the vertical jump measurement [22,23,24].
- Prowler Push—The athletes performed the prowler push assessment using a prowler sled loaded at a competition weight. All athletes remained within 5% of competition weight during testing. The sled was pushed for 15 meters as fast as possible with maximal effort. Push sled times were recorded for total distance [25].
- Modified Bruce Protocol (VO_2_max)—The athletes performed a progressive, maximal treadmill test to volitional exhaustion, using the Modified Bruce Protocol. This consists of increasing treadmill speed and incline. The test was terminated when the athlete could no longer continue. The maximal heart rate was recorded as the highest heart rate achieved during the test [26,27,28,29,30,31,32].
- Heart Rate Recovery [(HRR) following VO_2_max testing - one minute done in %]—Heart rate was measured throughout VO_2_max testing and for 1 minute during recovery following test completion [31].
- Pull Ups—The athletes performed the pull-ups until reaching a failure test using a horizontal bar with a prone-grip. The athletes pulled up with a straightened-body, high enough for the chin to be above the bar. The test terminated once the athlete was not able to clear the bar, started waving legs, or when the athlete hung longer than two seconds without being able to clear the chin above the bar. This motion was repeated, without rest, as many times as possible. Maximum repetitions were recorded [33,34,35].
- Injuries—The total number of injuries endured over the six-week training period were recorded.
- Missed Sessions—The total number of missed practices, competitions, and testing sessions due to injury were recorded.

### 2.3. Protocol Sleep Measures

All athletes were monitored continuously for sleep by using validated, wrist wearable technology (Readiband, Fatigue Science, Vancouver, BC, Canada) [36]. The following are based on the manufacturer’s description.

- Sleep Latency—The time it takes an individual to fall asleep.
- Sleep Efficiency—Indicates how much time in bed is spent sleeping.
- Onset and Wake Variances—Measures how consistent the athlete’s onset and wake times are.

### 2.4. Statistical Analysis

All descriptive statistics (means and standard deviation) were calculated for physical characteristics (e.g., height, weight, age), physical performance measures (e.g., vertical jump, VO_2_ max, prowler sled push, pull-ups), and sleep performance measures (e.g. sleep latency, sleep efficiency, onset, and wake variances). In addition, multiple correlation analyses were utilized to examine the potential relationships that may exist between sleep performance measures and physical performance measures. All statistics were analyzed using Statistical Analysis Software (SPSS, Version 21.0, IBM INC., Armonk, NY, USA) and the significance was set at P < 0.05.

## 3. Results

All descriptive data including age, body height, and body mass can be found for the group of male MMA athletes in Table 1. It should be noted that all athletes are relatively similar in body mass since MMA is a weight restricted sport. All athletes compete in the 77.1 kg (170 lbs.) weight division of the UFC. Table 2 will provide all of the results, which relate sleep with physical performance testing during the training. The correlation analysis assessing the relationship between sleep latency and performance testing measures revealed a strong significant (r = −0.860, P = 0.006) negative correlation between sleep latency and VO_2_max, sleep latency and heart rate recovery (r = −0.739, P = 0.036), sleep latency and prowler sled push times (r = −0.776, P = 0.024), sleep latency and vertical jump height (r = −0.787, P = 0.020), and, lastly, sleep latency and missed training sessions (r = −0.789, P = 0.035).

Additionally, the correlation analysis (Table 2) testing the relationship between sleep efficiency and performance testing measures revealed a significant (r = −0.891, P = 0.003) negative correlation between sleep efficiency and heart rate recovery.

Lastly, the correlation analysis (Table 2) that assess the relationship between Onset Variances and performance testing measures revealed a significant (r = −0.710, P = 0.049) negative correlation between the consistency in fall asleep time and heart rate recovery. The correlation analysis also revealed a significant (r = 0.788, P = 0.035) positive correlation between Onset Variances and missed practice sessions. Analysis revealed no other significant (P > 0.050) correlations between physical performance measures and sleep performance measures. 

## 4. Discussion

There are many questions with regard to sleep and performance among professional athletes. Travel schedules and sleep deprivation have shown to have detrimental effects in anaerobic athletes inlcuding power sports [16,19]. MMA is a hybrid sport in which anaerboic athletes travel across timezones in order to compete professionally. Because of the lack in literature and the lack in MMA research, we decided to present observational data with regard to sleep and physical performance. The results indicate that athletes slept, on average, more than seven hours per night. Previous literature has shown that seven to nine hours of sleep is desirable for proper human function and recovery [1]. 

Lack of sleep can expose athletes to detrimental effects regarding not only physical performance but also immune function, cognitive capacity, and psychomotor performance [37,38,39,40]. The findings demonstrate the relationships between the different sleep metrics and physical performance testing in MMA athletes. Sleep variance and sleep efficiency are two important metrics that demonstrate relationships with multiple variables including a decreased number in missed sessions and increased cardiovascular recovery. Sleep latency demonstrated relationships with improved performance measures among both aerobic and anaerobic states. Previous literature in both elite volleyball athletes and professional rugby players support the concept that sleep is a critical factor for the enhancement of athletic performance [41,42]. The data on sleep latency relating to decreased injury and missed sessions supports this notion. Research has also shown that a chronic lack of sleep correlates with increased injury risk, increased levels of depression, stress, and anxiety [43,44,45]. Our data supports this as the onset and wake variance relationships that exist with injury rates during the training.

Furthermore, an early-morning training session may greatly restrict the amount of sleep obtained by athletes [38]. In addition, previous reports show athletes with worse than usual sleep in the nights prior to an important competition [46]. Longer sleep durations have been associated with overall sport performance [37]. The effects of sleep extension on athletic performance including reaction time, mood, and daytime sleepiness have also been documented [47]. It is paramount that coaches and managers implement adequate scheduling to allow for both rest and recovery since a better regime may lead to improved physical performance and a decline in the injury rate [39]. Coaches and managers dictated all scheduling for MMA athletes during this six-week period. 

While the current study is one of the first to assess professional MMA fighters in a real-world (quasi-controlled) setting, it is not without limitation. First, the sample size was relatively limited even though access and resources to a large sample of professional MMA athletes is difficult due to injuries, fight schedule, and participation rates. The study design leaves for certain factors that may be uncontrolled such as injury timing and missed practices. These are uncontrolled as this was a “real-world” fight camp for the MMA athletes. These factors could also relate to the results of the physical testing not sleep alone. Future research should aim to assess different markers of fight performance such as striking and grappling statistics in competitive fights. 

## 5. Conclusions

In conclusion, it was hypothesized that consistent sleep metrics will relate to improved performance and health and professional MMA athletes. Based on the results, the ability to maintain a consistent sleep schedule may prove beneficial since they relate to performance improvements and injury reduction. Although sleep quality and quantity may be compromised during training camps, daytime rest may be beneficial for athletes [39,40,41,42]. Based on our study, the athletes who showed consistency in sleep also demonstrated superior performance testing throughout the six-week fight-camp period. Moreover, these athletes also missed significantly fewer sessions due to fatigue and injury throughout. This application may translate to other occupations that follow an unorthodox schedule of preparation and execution. For example, tactical occupations such as military, law enforcement, and fire departments may benefit from consistency in sleep metrics. Further research is currently underway. 

## Figures and Tables

**Table 1:** Participant demographics (means and SD).

MMA Athlete (n = 7)	**Age (years)**	**Body Height (cm)**	**Body Weight (kg)**
	27.2 ± 3.4	180.7 ± 4.4	77.3 ± 0.3

**Table 2:** Correlational data sleep, performance, and injury.

	Vertical Jump	Prowler Push	VO_2_max	HRR	Pull Ups	Injuries	Missed Sessions
Total Sleep Time							
r	0.191	−0.135	−0.126	0.191	0.669	0.155	−0.146
p	0.651	0.750	0.765	0.651	0.070	0.741	0.755
Sleep Latency							
r	−0.787 *	0.776 *	−0.860 **	−0.739 *	−0.370	−0.457	−0.789 *
p	0.020	0.024	0.006	0.036	0.366	0.303	0.035
Sleep Efficiency							
r	−0.137	0.539	−0.543	−0.891 **	−0.685	−0.628	−0.633
p	0.745	0.168	0.164	0.003	0.061	0.131	0.127
Onset Variances							
r	0.593	−0.640	0.472	0.710 *	0.219	0.682	0.788 *
p	0.121	0.087	0.238	0.049	0.602	0.091	0.035

* Denotes Significance p ≤ 0.05. ** Denotes Significance p ≤ 0.01.
